# Differential Activation of the ER Stress Factor XBP1 by Oligomeric Assemblies

**DOI:** 10.1007/s11064-012-0780-7

**Published:** 2012-04-21

**Authors:** Diana L. Castillo-Carranza, Yan Zhang, Marcos J. Guerrero-Muñoz, Rakez Kayed, Diego E. Rincon-Limas, Pedro Fernandez-Funez

**Affiliations:** 1 Department of Neurology, University of Texas Medical Branch, Galveston, TX 77555 USA; 2 Department of Neurology, McKnight Brain Institute, University of Florida, 1149 South Newell Dr., Gainesville, FL 32611 USA; 3 Departments of Neurology and Neurosciences, McKnight Brain Institute, University of Florida, 1149 South Newell Dr., Gainesville, FL 32611 USA

**Keywords:** Oligomers, Neurodegeneration, ER stress, XBP1, Amyloids

## Abstract

Several neurodegenerative disorders are characterized by protein misfolding, a phenomenon that results in perturbation of cellular homeostasis. We recently identified the protective activity of the ER stress response factor XBP1 (X-box binding protein 1) against Amyloid-ß1-42 (Aß42) neurotoxicity in cellular and *Drosophila* models of Alzheimer’s disease. Additionally, subtoxic concentrations of Aß42 soluble aggregates (oligomers) induced accumulation of spliced (active) *XBP1* transcripts, supporting the involvement of the ER stress response in Aß42 neurotoxicity. Here, we tested the ability of three additional disease-related amyloidogenic proteins to induce ER stress by analyzing *XBP1* activation at the RNA level. Treatment of human SY5Y neuroblastoma cells with homogeneous preparations of α-Synuclein (α-Syn), Prion protein (PrP106–126), and British dementia amyloid peptide (ABri1-34) confirmed the high toxicity of oligomers compared to monomers and fibers. Additionally, α-Syn oligomers, but not monomers or fibers, demonstrated potent induction of *XBP1* splicing. On the other hand, PrP106–126 and ABri1-34 did not activate *XBP1*. These results illustrate the biological complexity of these structurally related assemblies and argue that oligomer toxicity depends on the activation of amyloid-specific cellular responses.

## Introduction

Neurodegenerative diseases encompass a complex group of neurological disorders characterized by progressive and widespread neuronal cell loss. The most common neurodegenerative disorders are caused by abnormal protein deposition in the form of highly ordered intra- and/or extracellular amyloid fibers. Alzheimer’s disease (AD) and Parkinson’s disease (PD) are among the most prevalent proteinopathies, affecting up to 40 % of the elderly population [25, 33]. Upon autopsy, AD and PD are characterized by amyloid plaques rich in Amyloid-ß42 (Aß42) and Lewy bodies containing α-Synuclein (α-Syn), respectively. These large protein aggregates have a critical diagnostic value and were originally proposed to play a causative role in disease. However, a modern understanding of the role of amyloids in disease suggests that the large, fibrillar aggregates may not be directly responsible for neurodegeneration [7, 32]. Large amyloid aggregates have also been postulated to exert a defensive role by storing these toxic proteins in cellular structures such as the aggresome (Lewy bodies in PD and nuclear inclusions in polyglutamine diseases) or be inert byproducts of neuronal death (amyloid plaques) [34].

In contrast, soluble aggregates (oligomers) seem to correlate better with neurotoxicity in cellular and animal models [5, 23, 27, 37]. Oligomeric assemblies are dynamic structures that perturb many cellular processes, including mitochondria and energy metabolism, cell signaling, calcium homeostasis, oxidative stress and cell survival/apoptosis, and more by mechanisms not completely known [13, 22]. Oligomers can bind to receptors in synaptic membranes, including nicotinic and NMDA (*N*-Methyl-d-aspartate) receptors and RAGE (Receptor for advance glycation endproducts), causing synaptic dysfunction and perturbation in signaling pathways [30]. They can also form pores that integrate in the membrane and alter the transport of essential ions, which may be responsible for calcium dyshomeostasis [3, 11]. Finally, oligomers can be actively transported by endocytic mechanisms, resulting in intracellular accumulation and interaction with the protein quality-control mechanism, including chaperones and the proteasome [19]. So far, this new focus on oligomers has identified common neurotoxic mechanisms among different amyloidogenic proteins based on shared structures [13]. One problem with the idea that all amyloidogenic proteins form structurally related oligomers is explaining the different cellular vulnerability and unique molecular pathology of each disease. Thus, oligomeric assemblies from different proteins must encompass unique biological properties that explain the disease-specific phenotypes. However, little is known at this time about the biological differences among oligomers.

We have shown recently that overexpression of the ER stress response factor *XBP1* (*X*-*box binding protein 1*) rescued the toxicity of human Aß42 expressed in transgenic flies [6]. Conversely, reduction of the endogenous *XBP1* function by RNAi increased the Aß42 phenotype, supporting the physiological role of *XBP1* in the response to Aß42 neurotoxicity. *XBP1* is a key component of the unfolded protein response (UPR), a conserved protective mechanism against misfolded proteins in the ER [15]. Three independent sensors regulate the UPR: PERK, ATF6 and IRE1. Upon ER stress, IRE1 autophosphorylates and dimerizes, which activates its cytoplasmic RNase domain. Active IRE1 then cleaves the *XBP1* pre-mRNA in the cytoplasm, which removes a 26-nt intron that changes the reading frame in the second exon of *XBP1*. This unconventional splicing results in the production of the transcriptionally active (spliced) isoform XBP1s instead of the inactive (unspliced) isoform XBP1u. XBP1s induces the transcriptional upregulation of a large number of target genes that contribute to reduce protein misfolding in the ER [1]. In turn, activation of ATF6 results in a cleaved fragment with potent transcriptional activity that induces the expression of several key target genes, including *XBP1*. Among our observations, we found that Aß42 activated ER stress and induced the unconventional splicing of *XBP1* in both transgenic flies and rat pheochromocytoma cells (PC12) treated with Aß42 oligomers [6]. Since subtoxic concentrations of Aß42 oligomers induced robust *XBP1* splicing in PC12 cells, ER stress and *XBP1* splicing seemed rapid and efficient cellular responses to Aß42 neurotoxicity. Moreover, these observations suggested that *XBP1* splicing could be used as a sensitive assay to detect Aß42 neurotoxicity and, possibly, the toxicity of other oligomers linked to neurodegenerative diseases. The assay used so far to detect *XBP1* splicing consists on an RT-PCR with primers straddling the small intron that amplifies both isoforms, *XBP1u* and *XBP1s*. Then, the PCR products are digested with *Pst*I, which has a unique, evolutionarily conserved restriction site in the intron of *XBP1u*, allowing the diagnostic identification of *XBP1s* transcripts (resistant to *Pst*I). Using this approach Lee and col. identified unconventional *XBP1* splicing in the temporal cortex of AD patients, but not in the cortex of Tg2576 mice, an AD model with no cell loss [24]. Thus, activation of the IRE1-XBP1 pathway seems to correlate better with neuronal degeneration than with deposition of misfolded Aß42, making *XBP1* an attractive diagnostic tool for neurodegenerative conditions.

From these observations, we hypothesized that oligomers from other amyloidogenic proteins, but not monomers and fibers, should also induce *XBP1* activation. In this report, we asked three questions: (1) do other amyloidogenic proteins induce *XBP1* activation, (2) which quaternary conformations of the protein induces *XBP1* activation, and (3) what is the diagnostic potential of *XBP1* splicing at the RNA level? To answer these questions, we produced monomeric, oligomeric, and fibrillar preparations for three disease-related amyloids: full-length α-Syn; the amyloidogenic fragment of the Prion protein (PrP106–126), and ABri1-34, an Aß42-related peptide associated with familial British dementia (FBD). Here we report that α-Syn oligomers, similar to Aß42, induce strong *XBP1* activation in SH-SY5Y cells by a modified PCR procedure. Surprisingly, PrP106–126 and ABri1-34 did not induce *XBP1* splicing, although their oligomers were as toxic as α-Syn oligomers. Overall, these results confirmed that oligomeric assemblies of other amyloidogenic proteins can induce unconventional splicing of *XBP1*, although this is not a conserved activity of all amyloids.

## Materials and Methods

### Preparation of Monomers, Oligomers, and Fibers

Soluble oligomers were prepared as shown by Kayed [21] by dissolving 0.3 mg of Aß42 (MW 4514), α-Syn (MW 14,460), PrP106–126 (MW 1912), and ABri1-34 (MW 3935) (previously re-solubilized in acetonitrile: water 1:1 and lyophilized) in 200 μl of hexafluoroisopropanol (HFIP) for 20 min at room temperature. 200 μl of these solutions were added to 1,000 μl DD H_2_O in a siliconized Eppendorf tube for evaporation of the HFIP, resulting in pure monomers at 0.3 mg/ml. The samples were then stirred at 500 rpm using a Teflon coated micro stir bar for 24–48 h at 22 °C for formation of oligomers. Before using oligomers for cell treatment, the samples were sonicated to break incipient fibers. Fibrils were prepared as described above for oligomers, except they were stirred at room temperature for 6–9 days. Fibril formation was monitored by thioflavin-T fluorescence and UV light scattering. Once fibril formation was complete, the solutions were centrifuged at 14,000×*g* for 20 min, the fibril pellet was washed three times with double-distilled water and then resuspended in the desired buffer. The morphology was verified by negative stain electron microscopy using standard procedures [21]. For the treatment of cells, the molarity for each peptide was calculated based on the amount of monomer.

### Cell Culture, Oligomer Treatment, and Cell Toxicity

Human neuroblastoma SH-SY5Y cells (Sigma) were grown in DMEM supplemented with 10 % FBS and differentiated in 10 μM retinoic acid and 1 % FBS for 24 h. To investigate toxic effects of different monomer, oligomer, and fibrils preparations, SH-SY5Y cells were seeded at 6,000 cells/well, in black 96-well clear bottom microtiter plates (Corning). The next day, cells were treated with 2 μM of monomer, oligomers, or fibrils in triplicate. After 8 h of treatment, media containing oligomers was replaced with 100 μl of fresh media and 10 μl of alamar blue (Biosurce) per well. Fluorescence was measured at 530–560 nm excitation and at 590 nm emission, using a fluorescence ELISA plate reader (Polar-star Omega BMG Labtech). To collect enough RNA for *XBP1* analysis, cells were incubated in 6-well plates at 5 × 10^6^ cells/well. After differentiating for 24 h, we treated them with 10 μg/ml of tunicamycin for 6 h to induce UPR as positive control for XBP1s accumulation. For XBP1 analysis, the cells were seeded in 6-well plates, treated with the peptides as described above, and collected for RNA extraction after 8 h of treatment.

### RT-PCR and Primers

10 μg of total RNA isolated from cultured cells (RNeasy Mini kit, Qiagen) were subjected to RT-PCR using Superscript III First Strand (Invitrogen). For amplification of both isoforms of human *XBP1*, we used primers hxbp6F 5′-GGAGTTAAGACAGCGCTTGG-3′ and hxbp6R 5′-ACTGGGTCCAAGTTGTCCAG-3′. In all RT-PCRs, a 323 bp *GAPDH* fragment was amplified as internal control using primers: hGAPDH-1F 5′-CGAGATCCCTCCAAAATCAA-3′ and hGAPDH-1R 5′-GTCTTCTGGGTGGCAGTGAT-3′.

### Electrophoretic Analysis of XBP1 Splicing

The procedure to detect *XBP1s* by digesting the *XBP1u* isoform was described before [6]. Briefly, half of the RT-PCR reaction was digested with *Pst*I, which cleaves *XBP1u*, but leaves *XBP1s* intact. For implementation of the *XBP1* electrophoresis without the hybrid band, we tried different concentrations of formamide in the loading buffer based on the TAE/hot formamide agarose electrophoresis method [26]. Briefly, PCR products were mixed with deionized formamide at different concentrations (up to 90 %), 1/10 sample volume of 10 × loading dye (50 mM Tris–HCl, pH 7.6, 0.25 % bromophenol blue, 60 % glycerol) and ethidium bromide at a final concentration of 0.1 μg/μl. Samples were then denatured by heating at 95° for 5 min, immediately chilled on ice for 5 min and loaded on 1.5 % agarose TAE gels. We also tried other denaturing conditions including the addition of 8 M urea with and without 0.1 % SDS to the loading dye, heating, and loading on 1 M urea-agarose gels. The best results were obtained when amplicons were resolved directly on 6 % Novex TBE precast polyacrilamide gels (Invitrogen) at high voltage 15–20 V/cm, stained with ethidium bromide and visualized with an Eagle Eye II Imaging system (Stratagene).

### Purification and Sequencing of Hybrid

The hybrid band was cut out from an agarose gel, purified by Qiaex II gel extraction kit (Qiagen) and the cDNA fragment was sequenced using the forward primer: hxbp6F 5′-GGAGTTAAGACAGCGCTTGG-3′. The resulting sequence was blasted against human sequences, which provided the alignment of the first 57 nt. The rest of the alignment with the *XBP1u* intron and *XBP1s* exon 2 was done manually.

### Quantitation, Statistical Analysis and Image Processing

Gels from three independent PCR experiments were quantified by densitometry. Each band was quantified and then the *GAPDH* band was used for normalization of each lane. Then, the values for all *XBP1* bands were normalized against *XBP1u* in the untreated experiment (arbitrarily set to 100). For the S/U ratio, the value of *XBP1s* was divided by *XBP1u* in each treatment and normalized against the untreated sample (arbitrarily set to 1). For statistical analysis, we used *T* test with one degree of freedom and considered *p* < 0.05 as statistically significant.

## Results

### Detection of XBP1 Activation by RT-PCR

**Fig. 1:**
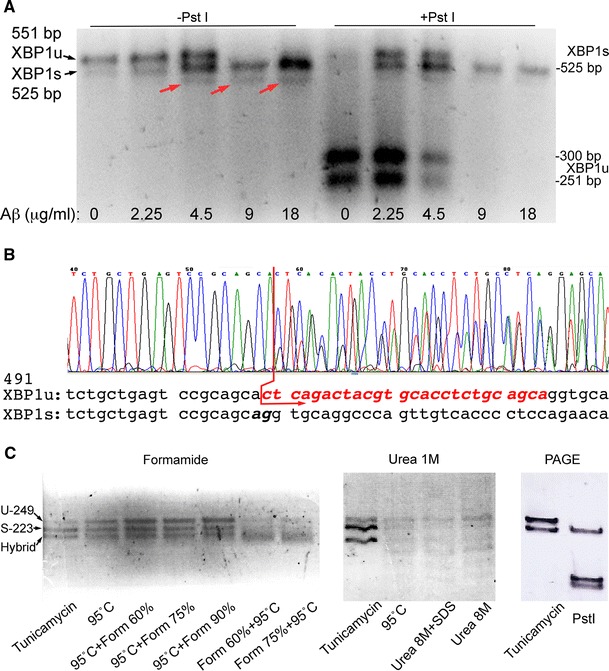
Detection of XBP1s by RT-PCR. **a** SY5Y cells treated with increasing concentrations of Aß42 oligomers induce *XBP1* splicing and accumulation of *XBP1s*, which is visualized by diagnostic digestion of *XBP1u* by *Pst*I. The non-digested samples that accumulate *XBP1s* contain a hybrid band that runs lower than *XBP1u* and *XBP1s* (*red arrows*). The *Pst*I-treated samples can help quantify *XBP1s* by digestion of *XBP1u*, but incomplete digestions complicate this effort. **b** Sequence of the hybrid band purified from an agarose gel. The sequence in the electropherogram matches both *XBP1* isoforms until it reaches the intron (*red arrow*). From this point, two overlapping peaks were detected corresponding to the *XBP1u* intron (*red sequence*) and *XBP1s* exon 2. **c** Elimination of the hybrid band in gel electrophoresis. Denaturing conditions such as formamide (*left*) and urea (*center*) did not eliminate the hybrid band even when combined with heat or SDS. The size for *XBP1u* (U) and *XBP1s* (S) is shown in the left gel. Resolving the PCR in polyacrylamide gels (PAGE) produced neat *XBP1s* and *XBP1u* bands. Upon digestion with PstI, a single *XBP1s* band was left (Color figure online)

As described above, *XBP1* activation can be easily detected by RT-PCR through the elimination of the 26-nt intron cleaved by IRE1 [6]. A detailed analysis of these gels showed that the accumulation of *XBP1s* was accompanied by the appearance of an extra band of slightly lower molecular weight than *XBP1s* (Fig. 1a, red arrows). This band was more prominent in conditions that favored *XBP1s* accumulation (treatment with Aß42 oligomers), suggesting that this was an artifact associated with the presence of two almost identical PCR products, possibly a hybrid *XBP1u/XBP1s* duplex. To verify that the extra band contained both *XBP1u* and *XBP1s*, we purified it from an agarose gel and sequenced it with the forward primer used for the RT-PCR. The first 57 nt of the sequence matched both isoforms, as expected, since the primer amplified a common region for both isoforms (exon 1) (Fig. 1b). However, starting with nt 58 the electropherogram revealed two clear overlapping peaks with half peak intensity in each position that indicated the co-existence of two sequences (Fig. 1b, red arrow). Reading the two peaks by eye, we identified the sequences corresponding to the alternative intron (*XBP1u*) and to exon 2 (*XBP1s*) (Fig. 1b). This result demonstrated that the extra band was a hybrid containing one copy of *XBP1u* and another of *XBP1s* that forms due to the almost identical sequence (except the 26-bp intron) of both PCR products.

Knowing the origin of the hybrid band still left the technical problem of quantifying the *XBP1u* and *XBP1s* isoforms. To eliminate the hybrid band during the electrophoresis, we tried several procedures, including denaturing conditions that prevent the formation of secondary structures in RNA and DNA gels. First, we tried a 60–90 % formamide gradient combined with heating up the sample to prevent the formation of the hybrids. Adding the formamide after heating the samples at 95 °C made the *XBP1u* band more stable, but did not eliminate the hybrid band (Fig. 1c, left). Heating the samples in the presence of formamide promoted DNA denaturation and resulted in weaker *XBP1u* and *XBP1s* bands (Fig. 1c, left). We also tested the effect of running the samples in 1 M urea agarose gels to prevent the formation of the hybrid. The urea gels resolved the bands neatly, but did not affect the hybrid (Fig. 1c, center). However, heating the samples at 95 °C and adding up to 8 M urea alone or in combination with 0.1 % SDS to the loading buffer produced unexpected effects on the samples that run in multiple weak bands (Fig. 1c, center). Finally, we tried 6 % Novex TBE polyacrylamide gels at high voltage and found that the hybrid band did not form in these conditions (Fig. 1c, right). When the PCR products were digested with *Pst*I, the *XBP1u* band split in two smaller products, leaving a single, neat band on top of the gel corresponding to *XBP1s* (Fig. 1c, right). It is not entirely clear why the hybrid disappeared in these conditions, although a number of factors could contribute to its instability, including the buffer and the heat generated by the high voltage. Regardless of the mechanism mediating the elimination of the hybrid band, these conditions resolving nicely the two *XBP1* isoforms allowed us to test the ability of other amyloidogenic proteins to induce unconventional splicing of *XBP1*.

### Oligomeric Assemblies are Highly Toxic

**Fig. 2:**
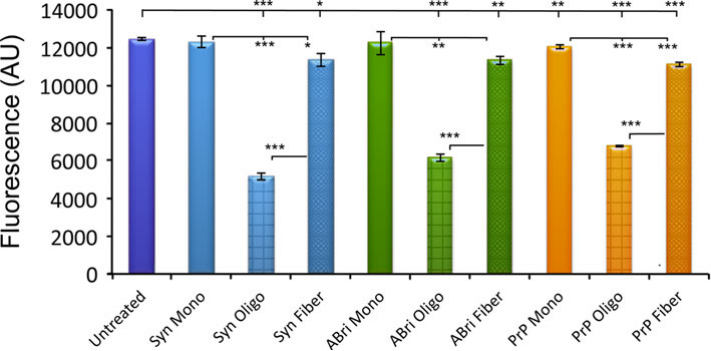
α-Syn, PrP106-126, and ABri1-34 oligomers induce cell death. Cell viability of SY5Y cells treated with oligomers, fibers, and monomers of α-Syn (*blue*), PrP106-126 (*orange*), and ABri1-34 (*green*) at 2 μM for 8 h. Oligomers for the three proteins (*squared patterns*) induced high toxicity resulting in around 50 % cell loss. Monomers (*solid colors*) did not affect cell viability, except for a slight, but significant reduction by PrP106-126. Fibers for each protein (*dotted*) reduced viability by about 10 %. *p < 0.05, **p < 0.01, ***p < 0.001 (Color figure online)

Before we began testing the ability of α-Syn, PrP106-126, and ABri1-34 to activate *XBP1*, we checked the cellular toxicity of each amyloid in different states of aggregation. For this, we generated monomeric, oligomeric, and fibrillar forms of α-Syn, PrP106-126, and ABri1-34 and added each into SY5Y cultures at 2 μM for 8 h in triplicate. The 2 μM concentration is based on our previous experience and extensive literature reporting a range of 500 nM to 2 μM for synthetic assemblies [5, 22]. Then, we determined cell viability using Alamar Blue, a compound that changes color when reduced inside living cells (Fig. 2). As expected, α-Syn monomers had no effect on viability, but oligomers reduced viability by almost 60 % (*p* < 0.0001, n = 3) (Fig. 2, blue). α-Syn fibers were slightly toxic, reducing viability by 9 %, but significantly less toxic than oligomers (*p* = 0.019, n = 3). Similarly, PrP106–126 oligomers were highly toxic to SY5Y cells, reducing viability by 46 % (*p* < 0.0001, n = 3) (Fig. 2, orange). PrP106–126 fibers were also slightly toxic (89 % viability, *p* < 0.0001, n = 3), but significantly less than the oligomers. Monomers reduced viability by only a few points (96.5 % viability), although this difference was significant (*p* < 0.005, n = 3), indicating the robustness and sensitivity of the assay. Finally, ABri1-34 showed the same trend as the other proteins, with highly toxic oligomers (49 % viability, *p* < 0.0001, n = 3) and slightly toxic fibers (91 % viability, *p* = 0.005, n = 3)) (Fig. 2, green). These results demonstrated that oligomers were highly toxic protein assemblies compared to the monomeric and highly aggregated forms. These observations revealed that all oligomers shared a common biological activity (cellular toxicity) regardless of sequence. Thus, these structures are ideal candidates to determine whether induction of ER stress and activation of *XBP1* unconventional splicing are common pathogenic mechanisms mediated by toxic oligomers.

### α-Synuclein Oligomers Activate XBP1 at Low Concentrations

PD is characterized pathologically by degeneration of dopaminergic neurons in the substantia nigra and the accumulation of protein aggregates in the cytosol known as Lewy bodies and Lewy neurites [25]. α-Syn is highly enriched in Lewy bodies and is thought to play a key role in PD neuropathology. At least three mutations in the coding region of *SNCA*, the α-Syn gene, as well as multiplications of *SNCA* lead to familial PD, supporting the strong role for α-Syn in PD [14]. α-Syn is a soluble presynaptic protein thought to exist in an unstructured state in solution, although a helical tetramer has recently been proposed to exist in physiological conditions [4]. α-Syn aggregates easily in vitro and its oligomers are very toxic to cultured neurons. However, α-Syn misexpression does not induce neurotoxicity in transgenic mice, leaving a big question about the role of α-Syn in the neurodegenerative cascade of PD.

**Fig. 3:**
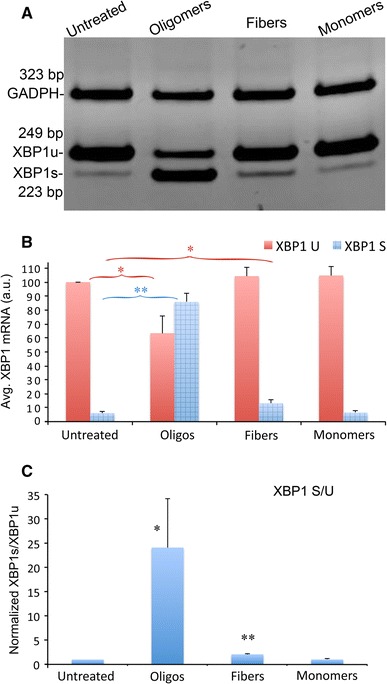
α-Syn oligomers are strong inducers of XBP1 splicing. **a** Unconventional *XBP1* splicing in SY5Y cells treated with α-Syn oligomers, fibers and monomers at 2 μM detected by RT-PCR. Untreated samples along with samples treated with monomers show high levels of *XBP1u* and very low levels of *XBP1s*. Samples treated with oligomers show the opposite, with higher levels of *XBP1s* than *XBP1u*. Samples treated with fibers show a subtle increase in *XBP1s*. **b** Quantitation of three independent experiments confirmed that monomers did not increase *XBP1s*, but fibers significantly increased *XBP1s*. However, oligomers had the largest effect by far, significantly reducing the levels of *XBP1u*. **c** The S/U ratio increases 24-fold in cells treated with α-Syn oligomers. Fibers double the S/U ratio, a significant difference with respect to untreated samples. α-Syn monomers do not affect the S/U ratio. *p < 0.05, **p < 0.01

To test the ability of α-Syn to induce *XBP1* activation, we treated SY5Y human neuroblastoma cells with 2 μM of monomeric, oligomeric, and fibrillar forms of α-Syn for 8 h. Then, we collected the cells, extracted total RNA and performed RT-PCR in triplicate to detect both *XBP1* isoforms with a set of primers spanning the intron. The reactions also contained primers for the housekeeping gene *GADPH* (*Glyceraldehyde*-*3*-*phosphate dehydrogenase*) for data normalization. Finally, we resolved the PCR products by PAGE as described above, quantified the results from each replicate, and normalized for statistical analysis. Figure 3a shows a representative experiment for *XBP1* activation by α-Syn preparations. As expected, non-treated cells accumulated mostly *XBP1u*, although a small amount of *XBP1* was detected slightly below. Approximately, 5 % of total *XBP1* is activated in normal conditions in the absence of ER stress in SY5Y cells (Fig. 3b), corresponding to constitutive expression of *XBP1s*, although the amount of constitutive *XBP1s* varies by tissue and cell line. In contrast, treatment with α-Syn oligomers resulted in prominent accumulation of *XBP1s* (*p* = 0.0016, n = 3) with the ensuing reduction of *XBP1u* (*p* = 0,037, n = 3) (Fig. 3a, b). In fact, the activation of *XBP1* was so strong that the *XBP1s* isoform became the most abundant band. Thus, α-Syn oligomers demonstrated a potent ability to induce the IRE1-XBP1 pathway.

As opposed to the dramatic effect of α-Syn oligomers, fibers and monomers had only subtle effects on *XBP1* splicing. Cells treated with fibers doubled the *XBP1s* levels, a much weaker effect than the oligomers, although the elevation of *XBP1s* was statistically significant (*p* = 0.027, n = 3) (Fig. 3a, b). However, no significant reduction on *XBP1u* was observed, suggesting that the increase in *XBP1s* was very modest. On the other hand, cells treated with monomeric α-Syn showed no significant change with respect to the untreated cells (Fig. 3a, b), supporting the idea that pure monomers are not toxic.

To evaluate more clearly the effect of α-Syn preparations on *XBP1* splicing, we calculated the ratio of *XBP1s* to *XBP1u* (S/U). The S/U ratio reversed dramatically from the untreated cells (normalized to 1) to the cells treated with oligomers (24, *p* = 0.022, n = 3), whereas cells treated with monomers remained unchanged (Fig. 3c). In cells treated with fibers, the S/U ratio doubled (2.06) and was statistically significant (*p* = 0.008, n = 3). Thus, both α-Syn oligomers and fibers induced *XBP1* splicing, indicating that both α-Syn assemblies are ER stressors. However, α-Syn oligomers were many times more potent inducers of *XBP1* splicing than fibers, supporting the unique ability of oligomers to perturb cellular processes, including ER stress, cell signaling, and cell death.

### PrP Oligomers do not Activate XBP1

Insoluble conformations of the PrP are associated with sporadic, genetic, and infectious forms of prion diseases [2]. PrP is a membrane-anchored glycoprotein highly expressed in the brain that is soluble in non-ionic detergents and easily digested by proteases. In its disease-associated ‘scrapie’ conformation, PrP is detergent insoluble, resistant to proteases and forms fibrillar aggregates [9]. PrP can misfold due to mutations in different domains, but sporadic forms of the disease show similar conformational changes as wild type PrP, indicating the intrinsic structural instability of PrP. Although much is known about the 3-dimentional structure of PrP and its conformational dynamics, it is still unclear how PrP causes neural loss and disease.

**Fig. 4:**
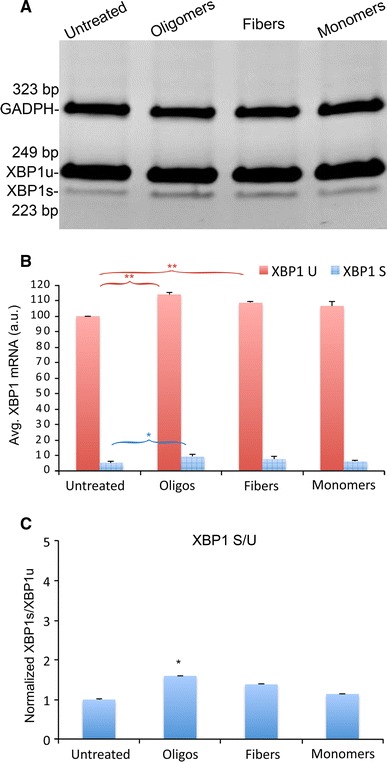
PrP106-126 oligomers are weak inducers of XBP1 splicing. **a** Unconventional *XBP1* splicing in SY5Y cells treated with PrP106-126 oligomers, fibers and monomers at 2 μM. All the samples had low levels of *XBP1s*, although both oligomers and fibers showed slightly stronger bands. **b** Quantitation of three experiments confirmed that *XBP1s* is significantly elevated only in samples treated with oligomers. Fibers induced a 50 % increase in *XBP1s* that was not statistically significant (*p* = 0.07). However, both oligomers and fibers induced the accumulation of higher levels of *XBP1u*. **c** The S/U ratio was mildly (less than double), but significantly elevated in cells treated with oligomers. *p < 0.05, **p < 0.01

For these studies, we used the PrP106–126 peptide to take advantage of its reported ability to induce neurotoxicity in cell culture [31]. This peptide contains the hydrophobic domain key for PrP fibrilization and forms amyloid fibers in vitro, while full-length PrP requires a tissue homogenate to do so, making it a less appropriate substrate for cell culture studies. As described above for α-Syn, we prepared homogenous PrP106–126 monomers, oligomers, and fibrils, treated SY5Y cells, and determined the activation of *XBP1*. In Fig. 2 we showed that PrP106–126 oligomers are as toxic as α-Syn oligomers. To our surprise, the effect of PrP106–126 assemblies on *XBP1* was very different from α-Syn. PrP106–126 oligomers at 2 μM showed poor induction of *XBP1* splicing (Fig. 4a). Upon quantification, we detected small, albeit significant, changes in the cells treated with oligomers and fibers, but not with monomers. PrP106–126 oligomers accumulated slightly higher levels of *XBP1s* (*p* = 0.017, n = 3), indicating weak induction of *XBP1* splicing (Fig. 4b). In addition, both PrP106–126 oligomers and fibers showed significantly higher levels of *XBP1u* (*p* = 0.0025 and *p* = 0.0021, respectively, n = 3) (Fig. 4a). This result indicated ER stress-dependent transcriptional activation of *XBP1*, which is typically mediated by the ATF6 sensor. PrP106–126 monomers, on the other hand, showed no significant changes in *XBP1* compared with the untreated controls (Fig. 4a, b), supporting the specific effects of PrP106–126 assemblies on XBP1 levels. The S/U ratio was low in all conditions, but the PrP106–126 oligomers showed a slight increase that was statistically significant (*p* = 0.027, n = 3). In summary, PrP106–126 oligomers weakly activated *XBP1* expression and splicing, demonstrating critical differences with α-Syn oligomers.

### ABri1-34 Oligomers Activate XBP1 at High Concentrations

Familial British dementia (FBD) is an autosomal dominant disorder characterized by progressive cognitive impairment and cerebellar ataxia. These symptoms are associated with amyloid deposition and neurofibrillary degeneration, sharing some similarities with AD [36]. FBD is linked to a mutation that eliminates the normal stop codon on *BRI2*, resulting in a longer precursor protein that generates a novel 34-residue amyloidogenic peptide named ABri1-34. Although ABri1-34 and Aß42 do not have sequence homology, they share many biological characteristics: both are secreted peptides that accumulate amyloid plaques in the brain and proposed to be the culprits in FBD and AD, respectively [35].

**Fig. 5:**
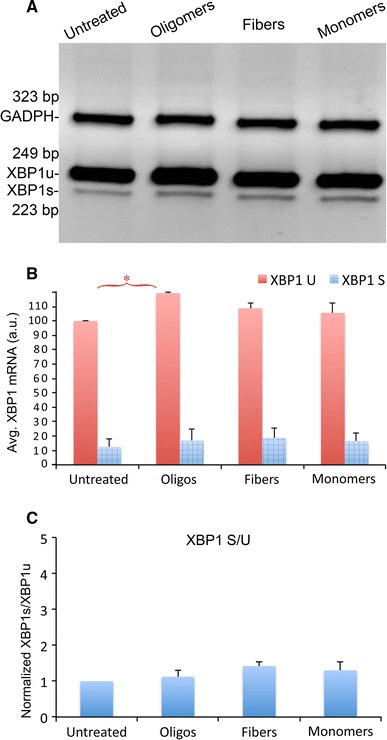
ABri1-34 oligomers are weak inducers of XBP1 splicing. (**a**, **b**) Unconventional *XBP1* splicing in SY5Y cells treated with ABri1-34 oligomers, fibers and monomers at 2 μM. All the samples revealed low levels of *XBP1s* with no statistical differences. However, the oligomers accumulated significantly higher levels of *XBP1u* as confirmed by quantitation. **c** The S/U ratio was not significantly altered in any of the samples. *p < 0.05

Once again, we prepared homogenous ABri1-34 monomers, oligomers, and fibrils, and treated SY5Y cells as described above to determine the activation of *XBP1*. Given the structural similarities to Aß42 and the toxicity of ABri1-34 oligomers, we were surprised to find that none of the ABri1-34 treatments induced *XBP1* splicing (Fig. 5a, b). However, ABri1-34 oligomers accumulated significantly higher levels of *XBP1u* (*p* < 0.001, n = 3) consistent with ER stress-mediated transcriptional activation of *XBP1*. Finally, the S/U ratio was not affected by the ABri1-34 treatments (Fig. 5c). These results indicated that ABri1-34 oligomers did not induce *XBP1* splicing in the same conditions in which Aß42 and α-Syn behave as strong inducers of *XBP1*, further indicating the different biological activity of these structurally related assemblies.

## Discussion

Neurodegenerative diseases are characterized by complex cellular perturbations involving synaptic, axonal, and mitochondrial dysfunction as well as transcription changes, among others. In contrast to these disruptive events, misfolded proteins can also launch adaptive, protective responses, including inflammation, Ubiquitin–Proteasome-dependent protein degradation, autophagy, and UPR. We are particularly interested in understanding the role of the UPR in disease because several recent studies have linked ER stress to some of the most prevalent neurodegenerative diseases, such as AD, PD, and amyotrophic lateral sclerosis (ALS) [28]. For instance, the brains of AD patients accumulate elevated levels of the ER chaperone Grp78/BiP, and phosphorylation of the UPR sensor PERK and its target eIF2α [8, 18]. In addition, the ER chaperone PDI and phospho-eIF2α are elevated in the brain of PD patients [10, 17] and in the spinal cord of ALS patients [20]. *XBP1* has only recently been used as a UPR marker based on the diagnostic value of the small intron regulated by the IRE1 sensor. *XBP1s* is elevated in the frontal cortex of AD patients, but not in mice expressing mutant APP [24]. We also showed that transgenic flies expressing human Aß42 and rat PC12 cells treated with Aß42 oligomers induce unconventional splicing of *XBP1* [6]. Moreover, reduction of endogenous *XBP1* increased Aß42 toxicity in flies, while *XBP1* misexpression ameliorated it [6]. In a chemical model of PD, mice treated with the toxin MPTP (1-methyl-4-phenyl-1,2,3,6-tetrahydropyridine) exhibited *XBP1* upregulation in the brain, while adenoviral expression of *XBP1s* protected dopaminergic neurons in these mice [29]. Mice inoculated with several strains of prions showed increased levels of the *XBP1s* isoform [16], suggesting the involvement of the IRE1-XBP1 pathway in PrP pathogenesis. These recent results suggest that *XBP1* is activated in tissues undergoing neurodegeneration and support the idea that *XBP1* activation is a neuroprotective response to amyloid insults. However, patient and animal studies are not ideal models to identify the conformations and assemblies directly responsible for inducing ER stress.

The purpose of the present study was threefold: (1) To develop a sensitive assay to detect *XBP1* activation at the RNA level, (2) compare the ability of several amyloidogenic proteins to induce *XBP1* splicing in the same experimental conditions, an (3) determine which assemblies are responsible for this activity. We show here that amplifying both *XBP1* isoforms by RT-PCR and running the PCR products on polyacrilamide gels eliminates the *XBP1u/XBP1s* hybrid, thus removing the main obstacle to exploiting RNA isoforms for diagnostic purposes. Our results demonstrate that changes in the relative abundance of *XBP1* isoforms are highly reproducible, supporting the use of RNA to accurately determine *XBP1* unconventional splicing. We are currently developing a quantitative PCR method to increase the sensitivity and precision for detecting *XBP1* splicing.

To answer the next two questions, we first confirmed that oligomeric preparations from α-Syn, PrP106–126, and ABri1-34 induced similar levels of cell toxicity (around 50 % lethality). On the other hand, monomers showed no toxicity at all and fibers induced a small but significant cell loss. These results support the idea that oligomers from different protein sources share unique biological properties that make them highly toxic. In contrast to the consistent cell toxicity of oligomers, the ability to induce *XBP1* activation was sequence-dependent. Of all the conditions tested, only α-Syn oligomers were potent inducers of *XBP1s*, resulting in a dramatic decrease in the levels of *XBP1u*. α-Syn fibers induced slightly higher levels of *XBP1s* than the untreated cells, but that effect was very modest compared to the oligomers. On the other hand, PrP106–126 and ABri1-34 assemblies were poor inducers of *XBP1* splicing. However, PrP106–126 and ABri1-34 oligomers induced a mild transcriptional upregulation of *XBP1u*, which could be due to the activation of other ER stress sensors, like ATF6, which is a known transcriptional regulator of *XBP1*. α-Syn may also induce transcriptional activation of *XBP1*, but since most of it is spliced, we do not appreciate an increase in *XBP1u*. These experiments uncover unexpected differences among amyloidogenic proteins, subdividing them into those that induce potent *XBP1* splicing (α-Syn, Aß42) and those that do not (PrP106–126, ABri1-34).

If the ability to induce *XBP1* splicing is highly dependent on specific structures only found in some oligomers, why did α-Syn fibers induce a slight activation of *XBP1*? There are two possible explanations for the mild effect of α-Syn fibers. One is that the fibril preparations may contain small amounts of pre-aggregated oligomers or that the oligomers are actively released from fiber breakage. This small amount of oligomers may explain the weak activation of *XBP1*, suggesting that fibers have no role in the induction of ER stress. Alternatively, highly pure fibrillar preparations may be directly responsible for *XBP1* activation, arguing for the preservation of oligomeric structures in the fibers that allow them to interact with the same cellular pathways.

Whereas all oligomers showed similar cell toxicity, a highly specific biological assay (*XBP1* activation) uncovered the contribution of the protein sequence to the activity of oligomers from four protein sources. The different ability of Aß42 and α-Syn oligomers to induce *XBP1* splicing compared to ABri1-34 and PrP106–126 oligomers support the existence of some degree of variation in the conformation of these two groups of oligomers. Unfortunately, it is unclear at this point what makes Aß42 and α-Syn capable of activating IRE1-XBP1 and why ABri1-34 and PrP106–126 do not. The available experimental evidence suggests that there may be little structural variation among the oligomeric conformations. This is supported by the ability of a few conformational antibodies to recognize multiple oligomeric species obtained from synthetic or biological sources and prepared by different methods [12]. These results argue for the existence of few stable conformations compatible with the formation of neurotoxic oligomers. Also, most oligomers show the ability to perturb membrane integrity and disrupt ion metabolism [11], pointing to common biological activities [13]. Since activation of UPR requires the perturbation of an internal organelle (the ER), exogenous Aß42 and α-Syn may be more efficiently transported into the ER by endocytic mechanisms. If this were the case, this would indicate the differential recognition of some oligomeric conformations, but not all, by specific receptors or transporters. Thus, we report here that XBP1 and the ER stress play different roles in neurodegenerative diseases, although the mechanisms underlying these differences are not clear. Additional structural approaches in the future may contribute to resolve in more detail the similarities and differences among these conformers critical in many chronic disorders.

Overall, we report here a strong connection of α-Syn to induction of ER stress and the XBP1-IRE1 pathway. Importantly, α-Syn misfolding and aggregation is an salient pathological feature of other neurological disorders, including dementia with Lewy bodies and multiple systems atrophy, suggesting that ER stress may be a common component of other synucleinopathies. Thus, identification of the signals that result in UPR and amelioration of this cellular response may contribute to the treatment of several synucleinopathies. On the other hand, there seems to be less consensus on the role of ER stress in prion diseases. The inability of PrP106–126 to induce *XBP1* splicing agrees with the observation that elimination of *XBP1* in mice did not alter the course of prion disease [16], suggesting that *XBP1* plays no physiological role in prion diseases. Finally, FBD is a rare dementia and little is known about its specific pathobiology. Our results indicate that despite the strong similarities between Aß42 and ABri1-34 (two small, secreted, amyloidogenic peptides that cause neurodegeneration), they may cause toxicity through different cellular pathways. In conclusion, we describe here the differential activation of *XBP1* by four amyloidogenic proteins, suggesting a complex involvement of UPR in disease, a pathway that in the last few years has been connected to a wide array of human maladies, including cancer, ischemia, and several chronic disorders [38].

## Abbreviations

Aß42: Amyloid-ß1-42
ABri1-34: British dementia amyloid peptide
AD: Alzheimer’s disease
FBD: Familial British dementia
PD: Parkinson’s disease
PrD: Prion disease
PrP: Prion protein
α-Syn: α-Synuclein
UPR: Unfolded protein response
XBP1: X-box binding protein 1

## References

[1] Acosta-Alvear D, Zhou Y, Blais A, Tsikitis M, Lents NH, Arias C, Lennon CJ, Kluger Y, Dynlacht BD (2007). XBP1 controls diverse cell type- and condition-specific transcriptional regulatory networks. Mol Cell.

[2] Aguzzi A, Sigurdson C, Heikenwaelder M (2008). Molecular mechanisms of prion pathogenesis. Annu Rev Pathol.

[3] Arispe N, Rojas E, Pollard HB (1993). Alzheimer disease amyloid beta protein forms calcium channels in bilayer membranes: blockade by tromethamine and aluminum. Proc Natl Acad Sci U S A.

[4] Bartels T, Choi JG, Selkoe DJ (2011). alpha-Synuclein occurs physiologically as a helically folded tetramer that resists aggregation. Nature.

[5] Benilova I, Karran E, De Strooper B (2012). The toxic Abeta oligomer and Alzheimer’s disease: an emperor in need of clothes. Nat Neurosci.

[6] Casas-Tinto S, Zhang Y, Sanchez-Garcia J, Gomez-Velazquez M, Rincon-Limas DE, Fernandez-Funez P (2011). The ER stress factor XBP1 s prevents amyloid-beta neurotoxicity. Hum Mol Genet.

[7] Caughey B, Lansbury PT (2003). Protofibrils, pores, fibrils, and neurodegeneration: separating the responsible protein aggregates from the innocent bystanders. Annu Rev Neurosci.

[8] Chang RC, Wong AK, Ng HK, Hugon J (2002). Phosphorylation of eukaryotic initiation factor-2alpha (eIF2alpha) is associated with neuronal degeneration in Alzheimer’s disease. NeuroReport.

[9] Colby DW, Prusiner SB (2011). Prions. Cold Spring Harb Perspect Biol.

[10] Conn KJ, Gao W, McKee A, Lan MS, Ullman MD, Eisenhauer PB, Fine RE, Wells JM (1022). Identification of the protein disulfide isomerase family member PDIp in experimental Parkinson’s disease and Lewy body pathology. Brain Res.

[11] Demuro A, Mina E, Kayed R, Milton SC, Parker I, Glabe CG (2005). Calcium dysregulation and membrane disruption as a ubiquitous neurotoxic mechanism of soluble amyloid oligomers. J Biol Chem.

[12] Glabe CG (2008). Structural classification of toxic amyloid oligomers. J Biol Chem.

[13] Glabe CG, Kayed R (2006). Common structure and toxic function of amyloid oligomers implies a common mechanism of pathogenesis. Neurology.

[14] Hardy J, Lewis P, Revesz T, Lees A, Paisan-Ruiz C (2009). The genetics of Parkinson’s syndromes: a critical review. Curr Opin Genet Dev.

[15] Hetz C (2012). The unfolded protein response: controlling cell fate decisions under ER stress and beyond. Nat Rev Mol Cell Biol.

[16] Hetz C, Lee AH, Gonzalez-Romero D, Thielen P, Castilla J, Soto C, Glimcher LH (2008). Unfolded protein response transcription factor XBP-1 does not influence prion replication or pathogenesis. Proc Natl Acad Sci U S A.

[17] Hoozemans JJ, van Haastert ES, Eikelenboom P, de Vos RA, Rozemuller JM, Scheper W (2007). Activation of the unfolded protein response in Parkinson’s disease. Biochem Biophys Res Commun.

[18] Hoozemans JJ, Veerhuis R, Van Haastert ES, Rozemuller JM, Baas F, Eikelenboom P, Scheper W (2005). The unfolded protein response is activated in Alzheimer’s disease. Acta Neuropathol (Berl).

[19] Hu X, Crick SL, Bu G, Frieden C, Pappu RV, Lee JM (2009). Amyloid seeds formed by cellular uptake, concentration, and aggregation of the amyloid-beta peptide. Proc Natl Acad Sci U S A.

[20] Ilieva EV, Ayala V, Jove M, Dalfo E, Cacabelos D, Povedano M, Bellmunt MJ, Ferrer I, Pamplona R, Portero-Otin M (2007). Oxidative and endoplasmic reticulum stress interplay in sporadic amyotrophic lateral sclerosis. Brain.

[21] Kayed R, Glabe CG (2006). Conformation-dependent anti-amyloid oligomer antibodies. Methods Enzymol.

[22] Kayed R, Head E, Thompson JL, McIntire TM, Milton SC, Cotman CW, Glabe CG (2003). Common structure of soluble amyloid oligomers implies common mechanism of pathogenesis. Science.

[23] Lambert MP, Barlow AK, Chromy BA, Edwards C, Freed R, Liosatos M, Morgan TE, Rozovsky I, Trommer B, Viola KL, Wals P, Zhang C, Finch CE, Krafft GA, Klein WL (1998). Diffusible, nonfibrillar ligands derived from Abeta1-42 are potent central nervous system neurotoxins. Proc Natl Acad Sci U S A.

[24] Lee JH, Won SM, Suh J, Son SJ, Moon GJ, Park UJ, Gwag BJ (2010). Induction of the unfolded protein response and cell death pathway in Alzheimer’s disease, but not in aged Tg2576 mice. Exp Mol Med.

[25] Lees AJ, Hardy J, Revesz T (2009). Parkinson’s disease. Lancet.

[26] Masek T, Vopalensky V, Suchomelova P, Pospisek M (2005). Denaturing RNA electrophoresis in TAE agarose gels. Anal Biochem.

[27] McLean CA, Cherny RA, Fraser FW, Fuller SJ, Smith MJ, Beyreuther K, Bush AI, Masters CL (1999). Soluble pool of Abeta amyloid as a determinant of severity of neurodegeneration in Alzheimer’s disease. Ann Neurol.

[28] Paschen W, Mengesdorf T (2005). Endoplasmic reticulum stress response and neurodegeneration. Cell Calcium.

[29] Sado M, Yamasaki Y, Iwanaga T, Onaka Y, Ibuki T, Nishihara S, Mizuguchi H, Momota H, Kishibuchi R, Hashimoto T, Wada D, Kitagawa H, Watanabe TK (2009). Protective effect against Parkinson’s disease-related insults through the activation of XBP1. Brain Res.

[30] Selkoe DJ (2002). Alzheimer’s disease is a synaptic failure. Science.

[31] Singh N, Gu Y, Bose S, Kalepu S, Mishra RS, Verghese S (2002). Prion peptide 106-126 as a model for prion replication and neurotoxicity. Front Biosci.

[32] Terry RD (1996). The pathogenesis of Alzheimer disease: an alternative to the amyloid hypothesis. J Neuropathol Exp Neurol.

[33] Thies W, Bleiler L (2011). Alzheimer’s disease facts and figures. Alzheimers Dement.

[34] Truant R, Atwal RS, Desmond C, Munsie L, Tran T (2008). Huntington’s disease: revisiting the aggregation hypothesis in polyglutamine neurodegenerative diseases. FEBS J.

[35] Tsachaki M, Ghiso J, Efthimiopoulos S (2008). BRI2 as a central protein involved in neurodegeneration. Biotechnol J.

[36] Vidal R, Frangione B, Rostagno A, Mead S, Revesz T, Plant G, Ghiso J (1999). A stop-codon mutation in the BRI gene associated with familial British dementia. Nature.

[37] Walsh DM, Selkoe DJ (2004). Deciphering the molecular basis of memory failure in Alzheimer’s disease. Neuron.

[38] Yoshida H (2007). ER stress and diseases. FEBS J.

